# Genetic Variability of the Grey Wolf *Canis lupus* in the Caucasus in Comparison with Europe and the Middle East: Distinct or Intermediary Population?

**DOI:** 10.1371/journal.pone.0093828

**Published:** 2014-04-08

**Authors:** Małgorzata Pilot, Michał J. Dąbrowski, Vahram Hayrapetyan, Eduard G. Yavruyan, Natia Kopaliani, Elena Tsingarska, Barbara Bujalska, Stanisław Kamiński, Wiesław Bogdanowicz

**Affiliations:** 1 School of Life Sciences, University of Lincoln, Brayford Pool, Lincoln, United Kingdom; 2 Museum and Institute of Zoology, Polish Academy of Sciences, Warszawa, Poland; 3 Stepanakert Branch of the Armenian National Agrarian University, Stepanakert, Armenia; 4 Scientific Centre of Zoology and Hydroecology, National Academy of Sciences of Armenia, Yerevan, Armenia; 5 Institute of Ecology, Ilia State University, Tbilisi, Georgia; 6 BALKANI Wildlife Society, Sofia, Bulgaria; 7 Department of Animal Genetics, University of Warmia and Mazury, Olsztyn, Poland; 8 Department of Cell and Molecular Biology, Science for Life Laboratory, Uppsala University, Uppsala, Sweden; Università degli Studi di Napoli Federico II, Italy

## Abstract

Despite continuous historical distribution of the grey wolf (*Canis lupus*) throughout Eurasia, the species displays considerable morphological differentiation that resulted in delimitation of a number of subspecies. However, these morphological discontinuities are not always consistent with patterns of genetic differentiation. Here we assess genetic distinctiveness of grey wolves from the Caucasus (a region at the border between Europe and West Asia) that have been classified as a distinct subspecies *C. l. cubanensis*. We analysed their genetic variability based on mtDNA control region, microsatellite loci and genome-wide SNP genotypes (obtained for a subset of the samples), and found similar or higher levels of genetic diversity at all these types of loci as compared with other Eurasian populations. Although we found no evidence for a recent genetic bottleneck, genome-wide linkage disequilibrium patterns suggest a long-term demographic decline in the Caucasian population – a trend consistent with other Eurasian populations. Caucasian wolves share mtDNA haplotypes with both Eastern European and West Asian wolves, suggesting past or ongoing gene flow. Microsatellite data also suggest gene flow between the Caucasus and Eastern Europe. We found evidence for moderate admixture between the Caucasian wolves and domestic dogs, at a level comparable with other Eurasian populations. Taken together, our results show that Caucasian wolves are not genetically isolated from other Eurasian populations, share with them the same demographic trends, and are affected by similar conservation problems.

## Introduction

The grey wolf *Canis lupus* is a top predator in terrestrial ecosystems of the Holarctic, and all aspects of its biology have been extensively studied (see [1] and [2] for review). However, the geographic distribution of these studies has been considerably biased, with the majority being focused on North American and European wolves. Studies on wolves from Asia have been relatively rare (except for studies on dog domestication, which were not explicitly focused on wolves), but they substantially contributed to our knowledge on this species. For example, two distinct mtDNA lineages were discovered in India and Himalaya [3]–[5] that are basal to other grey wolf lineages worldwide. Another distinct lineage was discovered in Japan [6]. Studies on morphological diversity also identified a variety of distinct types of grey wolves in Asia, which constituted a basis for subspecies delimitation (see [7] for review).

The Caucasus is situated at the geographic border between Europe and Asia, with the Greater Caucasus Mountain Range constituting both biogeographic and political borders. This region is situated within the continuous distribution of the grey wolf and links populations from European Russia with these from the West Asia (Middle East). Despite the range continuity, considerable morphological variability has been reported for grey wolves in this region [7]. Caucasian wolves have been assigned to a distinct subspecies *C. l. cubanensis* Ognev, 1923 [8]. North of the Caucasus, the nominal subspecies *C. l. lupus* occurs, with a widespread range throughout Eastern Europe and North Asia [7]. South of the Caucasus, another subspecies, *C. l. pallipes* Sykes, 1831 [9], has been described, with the range covering south-west Asia from Turkey and Israel to India [10].

In some regions, grey wolf subspecies defined based on morphological differentiation display high level of genetic distinctiveness, e.g. Iberian wolves *C. l. signatus* Cabrera, 1907 [11] and Italian wolves *C. l. italicus* Altobello, 1921 [12] are genetically distinct from Eastern European wolves (*C. l. lupus*) [13]–[15]. Therefore, based on the morphological distinctiveness of the Caucasian wolves it may be expected that they will be genetically distinct from the neighbouring Russian and Middle Eastern wolf populations. On the other hand, there are also cases where morphological delimitation of subspecies is inconsistent with the genetic data – for example, Himalayan wolves belong to a mtDNA lineage that is unique for this region and distinct from other Eurasian wolf lineages [3]–[5], but based on morphology they have been classified as a subspecies *C. l. chanco*, which has a wide range throughout China and Mongolia.

Little is known about population history of wolves from the Caucasus and their genetic relatedness to other Eurasian wolf populations. The Caucasus region is considered as an important glacial refugium for temperate species of plants and animals, alongside with the Iberian, Apennine and Balkan refugia in Europe [16]–[18]. However, the grey wolf is not a typical temperate species. It occurred north of the European glacial refugia during the Late Pleistocene [19] and constituted a part of the Pleistocene steppe fauna [20], so the importance of glacial refugia for evolutionary history of this species is uncertain. Contemporary patterns of mtDNA haplotype distribution are inconsistent with the phylogeographic division into the glacial refugia; instead, two main haplogroups overlap spatially throughout Eurasia, and their geographic origins are unclear [21]. Mitogenome sequencing of ancient wolf-like canids revealed another, ancient lineage, but it was only detected in three late Pleistocene specimens from Belgium and has not been found in contemporary wolves [22]. One of the contemporary haplogroups, Haplogroup 2, was frequent in European wolves in the late Pleistocene and early Holocene, but its frequency subsequently declined. Contemporarily, Haplogroup 2 is abundant in Italy, the Balkans and the Carpathian Mountains and rare elsewhere in Europe [21]. In Asia, only four mtDNA haplotypes identified up to now belong to this haplogroup [21], and two of them occur in the Middle East [23]. Haplotype composition of the Caucasian wolves may therefore shed light on the phylogeographic history of wolves in Eurasia.

A recent study by Kopaliani *et al*. [24] revealed that grey wolves in Georgia (southern Caucasus) share common mtDNA haplotypes with the free-ranging domestic dogs from this region (livestock guarding dogs and mongrel dogs). This was interpreted to be a result of a recent hybridisation, and the analysis of nuclear microsatellite loci revealed that 13% of wolves and over 10% of dogs have an admixed ancestry [24]. Gene introgression from dogs may bias the interpretation of phylogeographic patterns and the reconstruction of evolutionary history of wolves in the Caucasus and globally. However, back-crossing into the wolf population may be dominated by hybrids mothered and raised by female wolves, which has no effect on mtDNA composition. Therefore, the shared mtDNA haplotypes are more likely to originate from contemporary wolves rather than dogs (while ultimately they all derive from ancient wolves).

In this study, we analyse genetic variability of the grey wolves from the Caucasus in comparison with other populations from Europe and the Middle East to assess whether their morphological distinctiveness is reflected in the genetic variability. We also reconstruct past demographic changes in the Caucasian wolves to evaluate whether or not they follow the trends reconstructed for European wolf populations [15] in order to understand whether the Caucasian wolves constitute a demographically independent population. In addition, we further address the issue of hybridization between wolves and other free-ranging large canids in the Caucasus.

## Materials and Methods

### Study Area and Sample Collection

**Figure 1:**
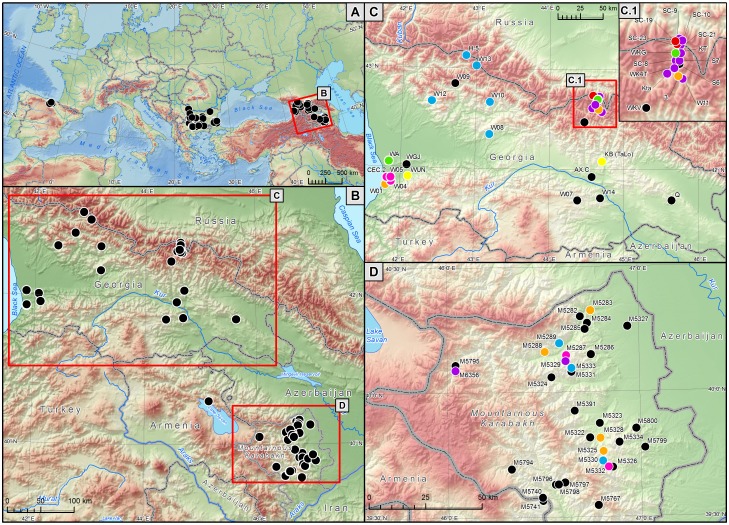
(A) Geographical location of the grey wolf population from the Caucasus in relation to Bulgarian and Spanish populations analysed for the comparative purposes; (B) Distribution of the samples in the Caucasus; (C) Distribution of closely related individuals identified in among wolves sampled in Georgia and (D) in Nagorno-Karabakh. Each colour represents one group of kin, and individuals without sampled kin are marked in black.

Our main study area was the south Caucasus, but – for comparative purposes – we also analysed grey wolf populations from Bulgaria and Spain (Figure 1A). Within the south Caucasus, we focused on two regions – Georgia and Nagorno-Karabakh. Georgia is situated in the south-western Caucasus on the southern slopes of the Great Caucasus mountain range (Figure 1B). About two thirds of the country is mountainous – the average altitude is 1,200 m above sea level (a.s.l.), with the highest altitude being 5,184 m a.s.l. Most wolf samples from Georgia originated from three regions: Kazbegi, Svaneti and Colchis. Kazbegi and Svaneti regions are situated in eastern and western parts of the Central Great Caucasus mountain range. The Colchis region is situated on the lowland plains in eastern Georgia that extend to the Black Sea. Average human density in Georgia is 65.4/km^2^.

Nagorno-Karabakh is located at the southeastern range of the Lesser Caucasus Mountains (Figure 1B). Most of the region is mountainous, and human density is low – 29/km^2^. Even in the lowlands human density is relatively low, and there are large areas uninhabited by humans. Most wolf samples were collected from three regions: Martakert, Kashatakh and Hadrut. Martakert and Kashatakh regions are mountainous, with high mountain peaks reaching 3,000–3,500 m a.s.l. Hadrut region is mountainous in the north-eastern part, while in the southern part there are lowlands of the river Araks, covered by meadows and pastures.

The natural vegetation in the Caucasus varies with altitude: the zone below 1,800 m a.s.l. is covered by deciduous, mixed and coniferous forests, the zone between 1,800 and 3,000 m a.s.l. is covered by subalpine and alpine vegetation, and high peaks are covered with glaciers. Nagorno-Karabakh has also semidesert and desert zones in some lowland areas. The semidesert biome is also present in the plains of eastern Georgia.

In Georgia, the potential wild ungulate prey of wolves include red deer (*Cervus elaphus maral*), roe deer (*Capreolus capreolus*), wild boar (*Sus scrofa*), and chamois (*Rupicapra rupicapra*). Turs (*Capra cylindricornis* and *Capra caucasica*) and bezoar goat (*Capra aegagrus*) inhabit high altitudes and rarely become wolf prey [25]. In Nagorno-Karabakh, the ungulate community is relatively different, as the wild sheep (*Ovis ammon*) is present, while two species of turs (*C. caucasica* and *C. cylindricornis*) are absent. Chamois and wild sheep are rare, and bezoar goat *C. aegagrus* is more abundant. In some areas (e.g. Colchis lowland and Kazbegi region in Georgia), domestic ungulates (cows, sheep, goats, donkeys, horses) are the main wolf prey. The most common non-ungulate prey in the south Caucasus is brown hare *Lepus europaeus,* and in Nagorno-Karabakh also porcupine *Hystrix leucura*. Besides the grey wolf, two other species of wolf-like canids occur in the south Caucasus, golden jackals (*Canis aureus*) and free-ranging domestic dogs (*Canis lupus familiaris*) [E. G. Yavruyan, unpublished data].

We obtained 65 samples of grey wolves from the south Caucasus: 33 from Georgia, 31 from Nagorno-Karabakh and one from Armenia (Figure 1B). The majority of samples (*n* = 50; 77%) were muscle or skin tissues. The remaining samples (from Georgia and Armenia) were hair (*n = *7; 11%) and faeces (*n* = 8; 12%). The muscle and skin tissue samples were obtained from individuals legally killed by local hunters. In Georgia wolf hunting is prohibited in general, but permissions are being given to kill particular individuals or packs involved in attacks on livestock. The samples were collected between 2008 and 2012.

We also analysed grey wolf tissue samples from Bulgaria (belonging to the nominate subspecies *C. l. lupus*; *n = *124) and Spain (*C. l. signatus*; *n* = 12), in order to directly compare their genetic variability with the Caucasian wolves and estimate the level of their diversification. These samples were collected between 2000 and 2012. Ninety-two Bulgarian samples were already genotyped for the purpose of earlier studies [26], [27], while the remaining samples were genotyped in this study.

For the purpose of testing for a presence of admixed individuals among the Caucasian grey wolves, we included to the analysis 14 free-ranging domestic dogs and three golden jackals from Bulgaria, genotyped by Moura *et al*. [27] for the same microsatellite loci as in this study. We also genotyped one sample of golden jackal from the south Caucasus (Nagorno-Karabakh). Ideally, we should have used a large number of samples from free-ranging dogs and golden jackals from the same areas as grey wolves in this analysis, but we did not have access to such samples. Our dataset was sufficient to identify admixed individuals (F1/F2 hybrids) and exclude them from further data analysis, but for a detailed analysis of hybridisation patterns among wolf-like canids in the Caucasus, more extensive sampling is required.

### Ethics Statement

Tissue samples used in this study were obtained from individuals that were killed as a result of legal hunting, from road kills, or from individuals that died of natural causes. No animal was killed for the purpose of this study.

### Laboratory Procedures

DNA from tissue samples was extracted at the Museum and Institute of Zoology, Polish Academy of Sciences using Genomic Mini Kit (A&A Biotechnology, Poland). DNA from hair and faeces was extracted in Georgia using Qiagen DNeasy kits (Blood and Tissue Kit and Stool Kit, respectively). Hair and faecal samples were pre-screened based on DNA concentration and PCR amplification success rate in the earlier analyses [24], and only samples that previously resulted in successful PCR reactions were used in this study. These samples were processed in a dedicated laboratory, separately from the DNA extracts from tissues, to avoid contamination. Negative controls of the extractions and PCRs were used to monitor for contamination.

We analysed a 660 bp fragment of the mtDNA control region, corresponding to the fragment analysed in Pilot *et al*. [21] and using laboratory procedures described there. The haplotype symbols used here are consistent with that earlier study [21]. We also analysed 14 microsatellite loci, applying laboratory procedures described in Pilot *et al*. [26] and Moura *et al*. [27]. Microsatellite loci were divided into five groups that were amplified in multiplex PCR reactions, here defined by brackets: (FH2010, FH2017, FH2054, FH2088 [28]), (FH2079, FH2096 [28], VWF [29]), (FH2001 [28], C213 [30]), (C250, C253 [30]), and (C466, C642 [30], AHT130 [31]).

In cases when two or more loci from one multiplex failed to amplify, we repeated the multiplex PCR for this particular sample up to three times. If amplification of one locus from a multiplex failed, we carried out a separate (non-multiplex) PCR for that locus. Separate PCR reactions were also carried out if there were uncertainties regarding a particular genotype at any locus amplified in the multiplex (e.g., due to stuttering or large differences in signal intensity between two allele peaks). To estimate the rate of genotyping errors, we replicated the genotyping of all 14 loci for 19 samples from the Caucasus (8 tissue, 5 hair and 6 faecal samples) and 14 samples from Bulgaria. Based on these replicates, the estimated allelic dropout rate in the Bulgarian samples was 0.04 and in the Caucasian samples 0.02, 0.02 and 0.05 for tissue, hair and faecal samples, respectively. The estimated false allele rate (wrong allele size scored due to stuttering, PCR artefacts or human error) in the Bulgarian samples was 0.01 and in the Caucasian samples 0.01, 0.03 and 0.01 for tissue, hair and faecal samples, respectively. We were not able to produce more genotyping replicates for the non-invasive samples due to the limited volume of DNA extracts available. However, these samples were pre-selected based on earlier PCR success rate [24], so their quality was higher than average for this type of samples. Moreover, as a result of kinship analysis a large proportion of non-invasive samples was removed from the final dataset (see below).

Another potential source of errors in microsatellite analysis are null alleles, i.e. alleles that fail to amplify due to primer binding site mutations or other reasons leading to a consistent PCR failure for a particular allele. We tested for the presence of null alleles in each population studied, following the procedure described in Dąbrowski *et al*. [32]. We found large inconsistencies in the detection pattern of putative null alleles among populations and detection methods applied, and concluded that the observed pattern does not justify the exclusion of any locus from the data analysis (see Supporting Information for details).

### Analysis of Admixture and Population Structure in the Caucasian Wolves Based on Microsatellite Loci

We used the software Structure [33] to assess the potential admixture between Caucasian grey wolves and other wolf-like canids. We expected some level of admixture between wolves and domestic dogs, as it has been documented in Georgia [24] and elsewhere in Europe [34]–[40] and in the Middle East [41]. There is also some evidence for hybridization between wolves and golden jackals [27], and therefore we tested for this possibility as well. For the admixture analysis, we used the dataset consisting of all the Caucasian grey wolves sampled (65 individuals), the dogs and the jackals.

The software Structure was also used to assess the level of diversification among the Caucasian wolves, in order to test for the population structure within this region. This analysis was run for 43 unrelated and non-admixed Caucasian wolves.

For both datasets, Structure was run with three independent chains and for number of groups (*K*) between 1 and 10, with 1,000,000 replicates preceded by 100,000 burn-in. We used the admixture model with correlated allele frequencies and no prior population information. The results were assessed using Structure Harvester [42]. The most likely number of groups was assessed based on the likelihood and the Δ*K* method [43].

Genetic structure in the Caucasian wolves was further assessed using the spatial model implemented in Geneland [44]. We ran ten independent chains for *K* values between 1 and 10, with 1,000,000 replicates preceded by 100,000 burn-in.

### Kinship Analysis

We identified close kin (parent-offspring pairs and siblings) using the combination of two methods: parentage assignment method implemented in Cervus [45], and sibshib reconstruction method implemented in Kingroup [46]. Cervus was used to identify parent-offspring pairs, and from these results we could also infer siblings as individuals sharing the same parent. We only accepted parent-offspring pairs identified at 95% confidence level and with no more than one mismatching locus. Kingroup was used to cluster individuals into groups at three levels of relatedness: parent-offspring, full-siblings and half-siblings. We checked for consistency between the two methods in parent-offspring and sibling groups identified.

We also compared mtDNA haplotypes of individuals identified as the close relatives. A wolf pack typically consists of a mating pair and their offspring, so we expected that in family groups identified based on microsatellite genotypes, most individuals (except for the father) will share the same mtDNA haplotype. We also mapped the family groups to check the spatial distribution of their members. Pack members share a common home range, but identified family groups could also include individuals that dispersed from their natal packs.

The presence of close kin may bias the assessment of genetic variability and demographic patterns in the populations studied, and therefore all but one individual from each kin group were removed from further analyses. Whenever it was possible to infer most likely parents based on the Cervus results and mtDNA comparison, the parents (or one parent if another one was absent from the sample) were retained in the dataset, while their offspring was removed. However, in cases where family members were genotyped based on different types of samples, we eliminated individuals that were genotyped based on non-invasive samples and retained a family member that was genotyped based on a tissue sample.

The same procedure of identification and elimination of closely related individuals from the dataset was also carried out for the Bulgarian and Spanish wolf datasets. After the elimination of close kin, the dataset applied for further analyses included 43 individuals from the Caucasus, 74 from Bulgaria and 7 from Spain.

### Analysis of Genetic Variability and Demographic Patterns Based on Microsatellite Loci

For this set of unrelated, non-admixed individuals from the Caucasus, Bulgaria and Spain, we estimated the genetic variability based on microsatellite genotypes. The average number of alleles per locus, expected and observed heterozygosity, and F_IS_ were assessed using GenAlEx [47]. We used Genepop [48] to test for Hardy-Weinberg equilibrium.

Effective population size (*N_E_*) was estimated using two methods based on linkage disequilibrium, implemented in the packages LDNe [49] and NeEstimator [50]. The presence of a signature of a genetic bottleneck was assessed using the program Bottleneck [51]. We used the Stepwise Mutation Model (SMM) and Two-Phased Model (TPM) with 70% share of the Infinite Allele Model (IAM). A mode shift in allele frequencies was also assessed.

We also estimated the level of gene flow among the Caucasian, Bulgarian and Spanish wolf populations using the assignment test implemented in GenAlEx, and calculated pair-wise Fst between these three populations using the same program. In addition, we run Structure analysis for these three populations, with three independent chains and 1,000,000 replicates preceded by 100,000 burn-in. We used the admixture model with correlated allele frequencies and run two sets of analyses either with or without using prior population information. Caucasian and Bulgarian wolves may be connected by gene flow through intermediary populations from Russia, Ukraine and Romania. The Iberian population is expected to be in a complete genetic isolation as a result of a large spatial distance from other populations (Figure 1A) and long-term demographic isolation [15], [52].

### Analysis of Genetic Variability, Demographic Patterns and Population Structure Based on mtDNA

The mtDNA haplotypes of wolves from the Caucasus were compared with the haplotypes from other parts of Eurasia, using the dataset compiled from earlier studies by Pilot *et al*. [21]. For these comparisons, we used the dataset of 660 bp long sequences (*n* = 45), which allowed us to compare the sequences in their whole length as produced in this study, as well as a dataset of 230 bp long sequences (*n* = 947), which allowed us to compare the larger number of mtDNA sequences from Eurasia (see [21]). The phylogenetic relationships among the haplotypes were reconstructed using the median-joining network approach implement in the software Network [53]. Because the dataset from Pilot *et al*. [21] did not include the data on wolf mtDNA haplotypes published after 2010, we searched GenBank using the Blast procedure for matches between the haplotypes found in the Caucasus (Table 1) and grey wolf haplotypes published after 2010, as well as any dog haplotypes.

**Table 1:** Frequency of mtDNA haplotypes in the Caucasus, and distribution of these haplotypes in Eurasia.

Haplotype	GenBank accession no.	Frequency	Other locations
w4	FJ978010	0.200	Belarus, Ukraine, Russia, Romania, Bulgaria, Greece
w7C	KJ195895	0.025	unique for the Caucasus
w10B	FJ978020	0.250	Poland, Russia, Croatia, Bulgaria, Greece, Turkey
w29	KJ490942	0.050	Israel
w32A	KJ490944	0.100	Saudi Arabia, Iran, India
w32B	DQ480507	0.075	Saudi Arabia, Iran, India
w47	KJ490943	0.125	Iran
w76	KJ195896	0.050	unique for the Caucasus
w77	KJ195897	0.125	unique for the Caucasus

We used Arlequin [54] to calculate mtDNA haplotype diversity (Hd) and nucleotide diversity (π), as well as Tajima’s D and Fu’s Fs tests, and assess the mismatch distribution. Genetic structure at mtDNA in the Caucasian population was assessed using the software Geneland [44], using the model that incorporates information on geographical location of the samples. This analysis was run with 10 independent chains for 1,000,000 generations after 100,000 burn-in for number of groups (*K*) between 1 and 10.

### Reconstruction of Demographic Changes from Genome-wide SNP Data

We analysed four unrelated wolves from Nagorno-Karabakh for genome-wide single nucleotide polymorphisms (SNPs) using the CanineHD BeadChip system (Illumina). We obtained variability data for 167,989 autosomal SNPs with an even genome-wide distribution.

Heterozygosity estimates based on genome-wide SNP data depend on the filtering methods applied. For inter-population comparisons, most studies exclude loci that are non-variable at the level of the entire dataset studied, while here we assess only one population. In order to obtain heterozygosity estimates comparable with other studies, we used the published dataset of SNP genotypes (80,223 SNPs) of European wolves from the study by Stronen *et al*. [55] (obtained using the same CanineHD BeadChip system) in order to select the set of autosomal SNPs which are variable for the broad dataset of wolves, and not just for the Caucasian individuals we genotyped. We then further pruned our Caucasian dataset to remove loci with missing data for more than one individual, which resulted in 78,550 SNPs. Based on this dataset, we calculated observed and expected heterozygosity. The SNP selection and heterozygosity assessment were performed in PLINK [56]. The dataset from Stronen *et al*. [55] was used only for the SNP pruning purposes and was not used in any subsequent analyses, which were based only on the genotypes from the Caucasus obtained in this study.

Further analyses required the set of loci that were variable within the sampled Caucasian population. Therefore, we pruned the initial set of 167,989 SNPs, removing loci that were invariable among the sample set or had any missing data. This pruning resulted in 86,531 SNPs. For this dataset, we calculated pair-wise identity by state (IBS), which is a measure of relatedness level between individuals. We also used this dataset to assess patterns of linkage disequilibrium and infer past demographic changes. For this purpose, we applied the same methods as in Pilot *et al*. [15] in order to obtain results that can be compared with other wolf populations from Europe. We identified runs of homozygosity (ROHs), i.e. chromosomal fragments that are homozygous within individuals. We looked for homozygous fragments at least 100 kb long and spanning at least 25 SNPs. Long ROHs (>1 Mb) are indicative of autozygosity (i.e. homozygosity by descent), which may result from recent inbreeding or admixture. Shorter ROHs (<1 Mb) result from population processes that took place in more distant past [57].

For all pairs of autosomal SNPs with minor allele frequency MAF>0.15 and no missing data, we estimated linkage disequilibrium (LD) by calculating genome-wide pairwise genotypic association coefficient (*r* ^2^), which is a squared correlation in genotype frequencies between autosomal SNPs. The average *r* ^2^ coefficient was calculated for 21 physical distance classes ranging from 1.25 kb to 1 Mb, in order to estimate the distance at which *r^2^* decays below a value of 0.5. For *r* ^2^ values within each distance class, we assessed standard error using bootstrap procedure with 1000 replicates, performed in R [58], and calculated 95% confidence intervals.

Average *r* ^2^ value within a particular genetic distance class in Morgans (*c*) provides an estimate of effective population size *t* generations ago, where *t*≈1/(2*c*) [59]. Following earlier studies (e.g. [15], [60]) we assumed that 100 Mb = 1 Morgan. We estimated average *r* ^2^ values in 20 distance classes between 2.5 kb and 1 Mb (corresponding to 0.0025–1 cM). We used the same distance classes as in the LD decay analysis, but eliminated the smallest distance class. These distance classes represent demographic changes from 50 to 20,000 generations ago, or 150–60,000 years ago, assuming the 3-year generation time [61]. *N_E_* values for each time intervals were estimated from the equation E(*r* ^2^)  = 1/(1+4*N_E_ c*) +1/*n*, where *n* is the sample size [62]. Pilot *et al*. [15] found that there was little difference between *N_E_* values estimated for 19 versus 6 Italian wolves, which suggests that past demographic changes may be inferred with sufficient accuracy from genome-wide SNP data even for a small number of individuals. The error of *N_E_* estimates was assessed based on the standard error of *r* ^2^ estimates.

For a comparison, we also provided the ROH, *r* ^2^ and *N_E_* plots for different European populations from Pilot *et al*. [15], including isolated populations from Italy and Spain that went through long-term bottlenecks, and local populations from Eastern Europe that are larger and interconnected, and for which there is no evidence of large-scale bottlenecks. The number of identified ROHs depends on SNP density, which was larger in this study as compared with Pilot *et al*. [15]. Therefore, we could not compare the absolute count of ROH fragments between populations, but instead we compared the proportion of ROHs of different length, and the shape of the curve representing the relationship between the number of ROHs and ROH length.

## Results

### Admixture between Caucasian Wolf-like Canids

The Structure analysis of the dataset consisting of Caucasian grey wolves, dogs and jackals identified the subdivision into three clusters (*K* = 3) corresponding to the three species as the most likely genetic structure. The dogs were assigned to the dog cluster with the likelihood 0.96–0.99, and the jackals were assigned to the jackal cluster with the likelihood 0.94–0.99 (Table 2). The majority (75%) of Caucasian grey wolves were assigned to the wolf cluster with the likelihood above 0.95, and further 12.5% with the likelihood 0.90–0.95. We also looked at the assignment of the grey wolves to both the dog cluster and the jackal cluster.

**Table 2:** Assignment probabilities of individuals to the three genetic clusters estimated in Structure.

Individuals	Wolf cluster	Dog cluster	Jackal cluster	mtDNA haplotypes of admixed individuals	Probable status
grey wolves	0.904–0.993	0.004–0.083	0.003–0.025	-	-
domestic dogs	0.003–0.035	0.962–0.995	0.002–0.014	-	-
golden jackals	0.003–0.036	0.002–0.007	0.957–0.996	-	-
				gj1 (golden jackal)	
misidentified jackal	0.042	0.015	0.943	KJ490945	golden jackal
				w77 (wolf)	
admixed individual W13	0.294	0.699	0.008	KJ195897	F1/F2 wolf-dog hybrid
				w4 (wolf/dog)	
admixed individual W08	0.348	0.644	0.008	FJ978010, AB605514	F1/F2 wolf-dog hybrid
				w77 (wolf)	
admixed individual W10	0.668	0.326	0.007	KJ195897	F1/F2 wolf-dog hybrid
				w77 (wolf)	
admixed individual H-5	0.694	0.299	0.007	KJ195897	F1/F2 wolf-dog hybrid
				w4 (wolf/dog)	
admixed individual 5284	0.700	0.281	0.019	FJ978010, AB605514	F1/F2 wolf-dog hybrid
				w76 (wolf)	
admixed individual 5796	0.776	0.210	0.013	KJ195896	F2 wolf-dog hybrid
				w32A (wolf)	
admixed individual 5799	0.860	0.137	0.004	KJ490944	F2/F3 wolf-dog hybrid
				w10B (wolf)	
admixed individual W09	0.343	0.338	0.319	FJ978020	unknown

For admixed individuals, mtDNA haplotypes, the species they match with (see the comment in the Supplementary Information) and GenBank accession numbers, as well as probable admixture status are also provided. Haplotype w4 was found in both grey wolves and domestic dogs. ‘Misidentified jackal’ is an individual sampled as a grey wolf that clusters with golden jackals and carries a golden jackal mtDNA haplotype.

Eight individuals morphologically identified as grey wolves (12.5%) were assigned to the dog cluster with likelihood between 0.14 and 0.70 (Table 2; Figure S1 in File S1). Five of these individuals had the assignment probabilities to the wolf and dog clusters in proportions close to 0.3: 0.7, or reverse (Table 2), and therefore their status as first-generation hybrids or back-crosses was ambiguous. These individuals carried five different mtDNA haplotypes (Table 2), all of which were found in non-admixed grey wolves. Only one of these haplotypes (w4) was also reported in GenBank as a haplotype of a domestic dog (from Japan; GenBank accession no. AB605514). The admixed individuals were removed from the analyses of genetic variability and population structure.

Almost all grey wolves were assigned to the jackal cluster with the likelihood below 0.025. One individual was assigned to each of the three clusters with similar likelihood (0.32–0.34). The equal admixture level for the three species is unrealistic; such result may indicate that this individual is an outlier genetically distinct from Caucasian grey wolves, but it cannot be considered as an evidence for wolf-jackal hybridisation. Another individual that was morphologically assigned as grey wolf, was assigned to the jackal cluster with the likelihood 0.94, and its mtDNA haplotype (GenBank accession no. KJ490945) clustered with published golden jackal haplotypes with 99% similarity (AF184048 [63]: 575/577 match, AY289996 [4]: 610/619 match, AY289996 [4]: 606/615 match). This individual was excluded from the analysis of genetic variability of Caucasian wolves.

### Kinship Analysis

Among the Caucasian wolf samples, we identified 11 groups of close relatives (parents and offspring or full siblings). These results were highly consistent between Cervus and Kingroup analyses: >85% of parent-offspring pairs and siblings identified in Cervus were also identified in Kingroup as either parent-offspring or siblings. The identified kin groups consisted of 2–9 individuals, and in each group either all or all but one individual carried the same mtDNA haplotype, suggesting that these groups could be packs (with shared mtDNA between a mother and offspring).

This is particularly likely in the case of faecal samples from Georgia, which were selected from a larger set of faeces collected from small areas, the spatial distribution of which was consistent with borders of pack territories [Kopaliani N, Gurielidze Z, Tevzadze G, Shaqarashvili M, Qurkhuli T, *et al*., unpublished data]. However, in many cases the related individuals were sampled in distant geographical locations (Figure 1C, D), suggesting that some members of kin groups have dispersed from their natal packs. One of these kin groups included 4 out of 8 individuals with an admixed wolf-dog ancestry, suggesting that they represent one hybridisation event rather than 4 independent events.

### Genetic Diversity at Microsatellite Loci and Population Differentiation between Wolves from the Caucasus, the Balkans and the Iberian Peninsula

Genetic diversity in each wolf population was estimated for a dataset consisting of non-related and non-admixed grey wolves. In the Caucasus, the average number of alleles per locus and the expected heterozygosity was similar as in Bulgaria, but the observed heterozygosity was higher as compared with Bulgaria, and as a result the inbreeding coefficient F_IS_ was lower (Table 3). In Spain, all the diversity indices were lower as compared with the Caucasus and Bulgaria (Table 3).

**Table 3:** Genetic diversity in the Caucasian wolves in comparison with wolf populations from Southern Europe.

Region (*n* samples)	mtDNA				microsatellite loci					Source
	*n* haplotypes (sequence length)	Haplotype diversity	Nucleotide diversity	*n* alleles per locus (SE)	H_O_ (SE)	H_E_ (SE)	F_IS_ (SE)	*N_E_* (LDNe)	*N_E_* (Ne Estimator)	
Caucasus (43)	9 (660 bp)	0.87	0.012	7.8 (0.8)	0.690 (0.033)	0.749 (0.031)	0.068 (0.020)	100	88	this study
Bulgaria (74)	11 (660 bp)	0.87	0.016	7.9 (0.9)	0.624 (0.032)	0.749 (0.026)	0.167 (0.023)	164	145	this study
Spain (7)	2 (660 bp)	0.29	0.004	2.9 (0.2)	0.505 (0.055)	0.547 (0.035)	−0.030 (0.100)	-	-	this study
Spain (47)	3 (333 bp)	0.56	0.010	5.5	0.525	0.645	0.177	43	54	[52]
Italy (103)	1 (546 bp)	0	0	4.4	0.44	0.49	0.10	-	-	[13]
Italy (435)	-	-	-	4.1–5.4	0.59–0.62	0.59–0.64	0.018–0.037	-	-	[76]

The global test for Hardy-Weinberg equilibrium indicated heterozygote deficit in the Caucasian and Bulgarian populations (*P*<0.0001 in both cases), and no deviations from the equilibrium in the Spanish population (*P* = 0.19).

Pair-wise F_ST_ between Caucasian and Bulgarian wolves was relatively small (0.024) compared to pair-wise F_ST_ between each of these populations and Spanish wolves (0.107 and 0.103, respectively). The GenAlEx assignment test suggested ongoing exchange of individuals between Caucasian and Bulgarian populations (with 16 individuals mis-assigned), but no gene flow between these populations and Spanish wolves (Figure S2A in File S1). The Structure analysis performed without prior population information (Figure S2B in File S1) identified two Caucasian wolves that were assigned with higher probability to the Bulgarian population than to their own population, and further two individuals with the assignment probability to the Bulgarian population >0.2. Two Bulgarian individuals were assigned with higher probability to the Caucasian population than to their own population, for two other individuals assignment probabilities were close to equal (0.5∶0.5), and further five individuals had the assignment probability to the Caucasian population >0.2. Most individuals had non-zero admixture levels between all three populations, which likely resulted from their common ancestry rather than recent migration. The Structure analysis performed with prior population information, which is more suitable for identifying recent migration, identified only one mis-assigned individual, a Bulgarian wolf with high (0.74) assignment probability to the Caucasian population (Figure S2C in File S1). This individual was analysed in an earlier study [27], so we could exclude the possibility that this sample has been mislabelled. While a direct dispersal on such large distance is unlikely, this result may indicate immigration from a less distant Eastern European population that shows higher genetic similarity to Caucasian than to Bulgarian wolves.

### Mitochondrial DNA Variability of the Caucasian Wolves

**Figure 2:**
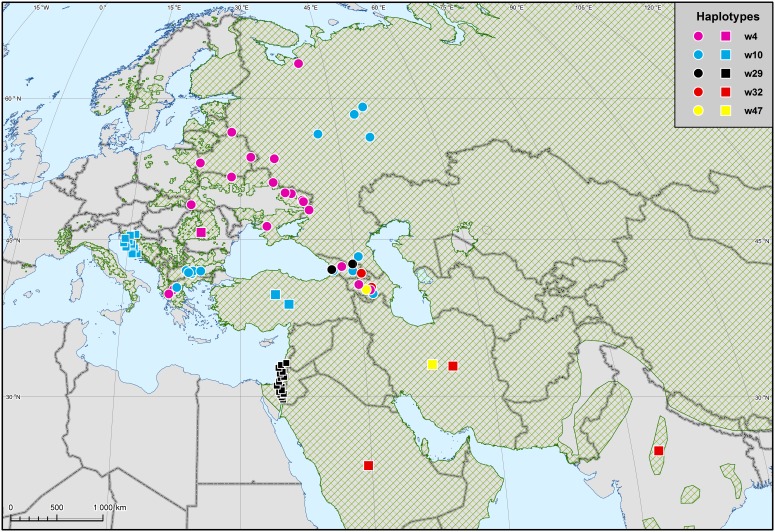
Distribution of mtDNA haplotypes found in the Caucasus and in other regions of Eurasia, against the background of the wolf range [77] **.** Samples from known localities are marked as circles, the origin of samples marked as squares is limited to the country range. Based on 230 bp sequence data.

**Figure 3:**
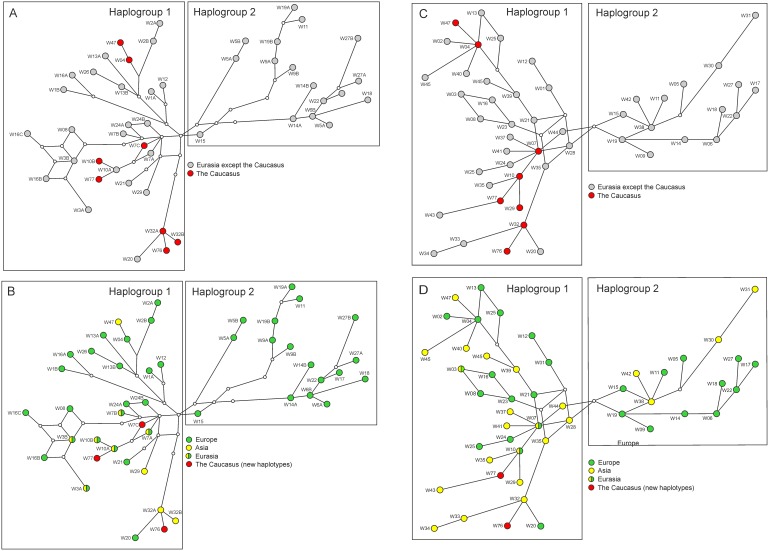
Median-joining network of mtDNA haplotypes constructed for 660 bp (A, B) and 230 bp long sequences (C, D). (A, C) Distribution of the Caucasian haplotypes (red) in the haplotype network; (B, D) Distribution of the haplotypes from Europe and Asia in the haplotype network. New haplotypes from the Caucasus are distinguished with different colour (red).

Based on 660 bp mtDNA control region sequence data, we found nine haplotypes in the Caucasian wolves. Three haplotypes (GenBank accession nos. KJ195895–KJ195897) have not previously been detected in any other region (and they have not been found in domestic dogs, either), so they may be unique for the Caucasus. Of the remaining six haplotypes, one has been found earlier in other parts of Europe, four in Asia, and one in both Europe and Asia (Table 1; Figure 2). Although one of these haplotypes, w4, has been also found in a domestic dog from Japan, this cannot be considered as an indicative of dog mtDNA introgression locally in the Caucasus, because this haplotype is widespread in European wolves (Figure 2). The Asian haplotypes were found in the Middle East and India; there were no common haplotypes with East Asia. Importantly, all the haplotypes found in the Caucasus belong to only one of two main wolf haplogroups (Haplogroup 1; Figure 3), while both haplogroups occur in other parts of Europe and Asia [21]. The Caucasian haplotypes were not phylogenetically clustered within Haplogroup 1, but instead were intermixed with haplotypes from different regions of Europe and Asia (Figure 3). Haplotype diversity (in a dataset consisting of non-related and non-admixed grey wolves) was estimated at 0.867±0.026, and nucleotide diversity at 0.0118±0.0062.

A comparison of Caucasian wolf mtDNA haplotypes with the combined datasets of wolf and dog haplotypes from the studies by Verginelli *et al*. [64] and Savolainen *et al*. [65] showed that two Caucasian haplotypes (w4 and w47) belong to a haplogroup shared between Eurasian wolves and domestic dogs, named haplogroup VI [64] or haplogroup B [65]. The remaining Caucasian haplotypes (including the three unique haplotypes) belong to a haplogroup containing most of worldwide wolf haplotypes and only one domestic dog haplotype, named haplogroup VIII [64] or haplogroup E [65].

The observed mismatch distribution for mtDNA haplotypes of the Caucasian wolves was inconsistent with the expected distribution for the demographic expansion model (Figure S3A in File S1). The sum of square deviations (SSD) index and the raggedness index (RI) were both significant (SSD = 0.066, *P*<0.0001; RI = 0.063, *P* = 0.027), indicating significant deviations from the sudden expansion model. For the spatial expansion model, both these indices were non-significant (SSD = 0.052, *P* = 0.07; RI = 0.063, *P* = 0.63), and the mismatch distribution was consistent with the expected distribution for this model (Figure S3B in File S1). Tajima’s D and Fu’s Fs tests were both non-significant (D = 2.24, *P* = 0.99; Fs = 4.52, *P* = 0.92).

### Population Genetic Structure of the Caucasian Wolves

We detected no population structure within the Caucasian wolves at microsatellite loci. Although the analysis with the software Structure (carried out for 43 unrelated, non-admixed grey wolves from the Caucasus) indicated *K* = 2 as the most likely genetic structure, assignment likelihoods to the two genetic clusters were close to even (i.e. about 0.5) for each individual, which indicated the lack of genetic structure. For higher *K* levels, assignment likelihoods to each genetic cluster were close to even as well. The spatially explicit model implemented in Geneland indicated *K* = 6 as the most likely population structure at microsatellite loci. However, the detected genetic groups were not geographically clustered, and all individuals showed high admixture levels between different clusters, with assignment likelihoods to the six genetic clusters close to even (i.e. about 0.17). Therefore, no spatial structure could be defined.

Similarly, for mtDNA data, running Geneland without accounting for spatial information indicated *K* = 2 as the most likely population structure, but the detected genetic groups were not geographically clustered. However, the spatially explicit model for mtDNA data showed a clear division into two genetically and geographically distinct groups, corresponding to Georgia and Nagorno-Karabakh, with the sample from Armenia and one sample from eastern Georgia grouping with Nagorno-Karabakh (Figure S4 in File S1). The assignment probabilities of individuals to their respective clusters were between 0.95 and 1, except for the individual from Georgia that was assigned to the Nagorno-Karabakh subpopulation with the probability 0.82.

### Demographic Reconstruction Based on Microsatellite Loci

Effective population size estimated in LDNe (using alleles with a frequency above 0.01) was 100 (95% CIs: 71–159) for the Caucasian wolves and 164 (95% CIs: 117–258) for the Bulgarian wolves. Effective population size estimated in NeEstimator was 88 (95% CIs: 68–119) for the Caucasian wolves and 145 (95% CIs: 114–195) for the Bulgarian wolves.

The Bottleneck test for the Caucasian population showed significant deviation from the mutation-drift equilibrium towards heterozygosity excess for the TPM model (Wilcoxon test: *P* = 0.001), but no deviation was detected when the SMM model was used (*P* = 0.91). The mode-shift test showed L-shaped allele frequency distribution expected for non-bottlenecked populations. Overall, these results do not provide a strong evidence for the occurrence of a genetic bottleneck. The bottleneck test for the Bulgarian population did not give clear results, either. The test for the TPM model showed significant deviation from the mutation-drift equilibrium towards heterozygosity excess (Wilcoxon test: *P* = 0.015), but no deviation was detected when the SMM model was used (*P* = 0.45), and the mode-shift test showed L-shaped distribution.

The demographic analyses were not carried out for Spanish wolves because the sample size was too small for this purpose. Instead, we used published *N_E_* estimates ([52], Table 3).

### Demographic Reconstruction Based on Genome-wide SNP Data

Pair-wise IBD values for the individuals analysed were between 0.55 and 0.67. This confirmed that these individuals were unrelated, as the threshold for individuals related at half-siblings or higher level was empirically established at IBD>0.8 for grey wolf populations [66]. The observed heterozygosity in the Caucasian wolves was estimated at 0.217, and the expected heterozygosity at 0.239.

**Figure 4:**
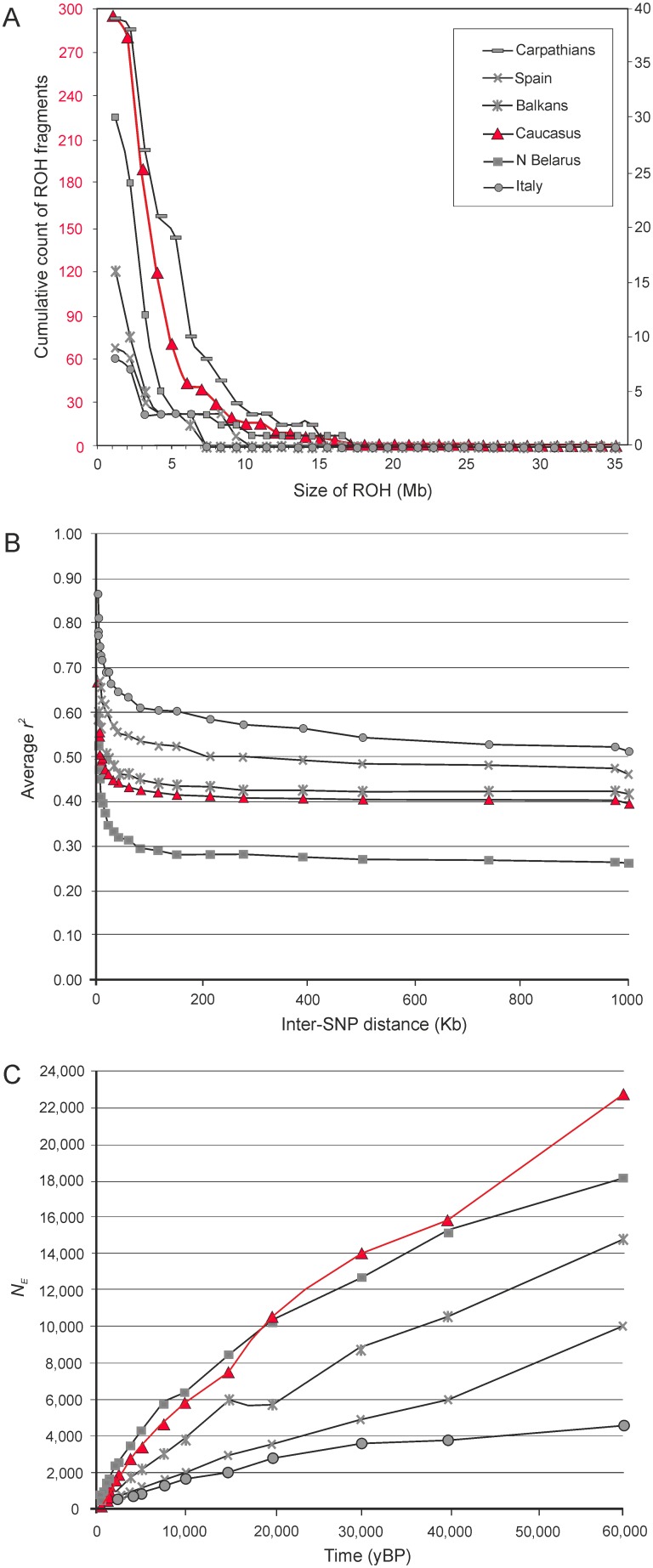
Inbreeding levels and demographic patterns in the Caucasian wolves inferred from genome-wide SNP data, in comparison with European wolf populations analysed in Pilot et al. [15]. (A) Frequency distribution of runs of homozygosity (ROH). This figure has different scales in the vertical axis for the Caucasus (left, marked in red) and other populations (right) because of differences in the number of SNPs analysed; (B) Extent of linkage disequilibrium, represented as changes in an average genotypic association coefficient *r^2^* with an increasing inter-SNP distance. (C) Temporal changes of effective population size (*N_E_*). We present the data for the populations representing extremes of the range (see Figure 5 in [15]), as well as the data for the Balkan and Spanish populations that were compared with the Caucasian population in other parts of this study. The Carpathian population is only presented in part A, because it had extreme values of ROH, but average values for other parameters.

The average number of homozygous segments per individual was 99, and their average length was 3506 Kb. ROH fragments <1 Mb long were relatively infrequent (26% of all ROHs), and the most frequent ROH fragments were between 1 and 5 Mb long (58%); the longest fragment was 34 Mb (Figure 4A). LD level was moderate (*r* ^2^ decayed below 0.5 at 7.5 Kb), as expected for a population that has not experienced a severe bottleneck (Figure 4B). The 95% confidence intervals for *r* ^2^ estimates delimited based on bootstrap standard errors are presented on Figure S5A in File S1. While we found no evidence for a recent bottleneck, the demographic reconstruction based on LD patterns showed that effective population sizes of Caucasian wolves gradually declined over the entire period considered (Figure 4C). The most ancient estimate of *N_E_* (at 60,000 years ago) was 22,750 (95% CI 20,098–24,913; Figure S5B in File S1), while the most recent estimate (at 150 years ago) was 144 (95% CI 140–148). These results suggest that the Caucasian population has experienced recent inbreeding (mating between individuals that have a recent common ancestor within few generations), which is not a result of a severe bottleneck, but a gradual population decline and other factors such as hunting pressure (see Discussion).

## Discussion

### Admixture between Caucasian Wolf-like Canids

Our results suggest a considerable level of admixture between Caucasian grey wolves and domestic dogs, consistent with the earlier study from Georgia [24]. All the admixed individuals had mtDNA haplotypes that also occurred in non-admixed grey wolves, suggesting that the prevalent mode of hybridisation leading to the backcrossing and gene introgression into the wolf population is between female wolves and male dogs. This is consistent with other studies on hybridisation between grey wolves and dogs in the wild [34]–[38], although reverse cases were also documented [39], [40]. Kopaliani *et al*. [24] found that over 10% of free-ranging dogs (mostly livestock guarding dogs) in Georgia have detectable wolf ancestry at microsatellite loci, which implies hybridisation between female dogs and male wolves, followed by the introgression of wolf genes into the dog population. According to Kopaliani *et al*. [24], besides spontaneous hybridisation, there may be cases of deliberate cross-breeding of these two species by humans: “in mountain parts of Georgia, dogs are occasionally paired with captured wolves, which allegedly ‘improves the breed’”. However, this process does not affect the genetic composition of the grey wolf population.

Among the mtDNA haplotypes found in Caucasian wolves, there were two haplotypes clustering with the domestic dog haplogroup B, as defined in [65], and one of these haplotypes (w4) was found in a domestic dog from Japan. However, both these haplotypes were detected in grey wolves in other parts of Eurasia (Figure 2), and therefore their clustering with dog haplotypes does not imply dog DNA introgression into the local wolf population in the Caucasus. Instead, it may be explained by a recent shared ancestry of these two canids (e.g. [22], [64], [65]), or introgression of wolf mtDNA into the dog population following hybridisation events in early phases of the evolutionary history of dogs.

The frequency of admixed individuals in the wolf population was relatively high (12.5%), and consistent with the corresponding assessment of the population from Georgia alone (13%; [24]). However, four of the eight admixed individuals were identified as close relatives, and therefore they cannot be considered as independent cases. After adjusting for this, the frequency of admixed individuals is reduced to 8%. For a comparison, the frequency of admixed individuals has been estimated at about 10% in Bulgaria [27], 5% in Italy [37] and 4% in the Iberian Peninsula [38].

We found no evidence for hybridisation between grey wolf and golden jackal in the Caucasus. However, there was one case of morphological misidentification of a golden jackal as a grey wolf, which was analogous to a case of three misidentified golden jackals reported in an earlier study from Bulgaria [27]. This may suggest the morphological similarity between these species in the areas of their co-occurrence, possibly resulting from some level of admixture. Another case of morphological misidentification has been described in North Africa, where canids that were previously classified as a subspecies of golden jackal where shown to carry mtDNA haplotypes of the grey wolf [67], [68]. Based on this finding it has been suggested to re-classify these canids as the African wolf *C. lupus lupaster*, although the authors also considered the possibility of hybridisation and gene introgression between different canid species [68]. The study from Bulgaria [27] found evidence for wolf-jackal hybridisation in that country, but at much lower frequency as compared to wolf-dog hybridisation. The occurrence and frequency of hybridisation may depend on the relative abundance of these three species and the hunting pressure on each of them, which varies among regions of their common distribution. A more extensive study covering the entire area of the range overlap between the grey wolf and the golden jackal is needed to understand the extent of their admixture and the conditions that may favour it. This is particularly important because of recent expansion of the golden jackal to the areas where previously the grey wolf was the only large wild canid (e.g. [69], [70]).

### mtDNA Variability of the Caucasian Grey Wolves in the Context of other Wolf Populations

The number of mtDNA haplotypes and haplotype diversity in the Caucasian wolves was similar as in the Bulgarian wolves (Table 3), which have high mtDNA diversity compared to other European populations [21], [27]. Nucleotide diversity in the Caucasian wolves was relatively low, which resulted from the fact that all the haplotypes found in the Caucasus belonged to Haplogroup 1 (as defined in Pilot *et al*. [21]), and Haplogroup 2 was not represented in the analysed sample. This was unexpected, because Haplogroup 2 is relatively frequent in Eastern Europe (13%; [21]) and also occurs in the Middle East (haplotypes w30 and w31 [21], [23]). Haplogroup 2 was common among European grey wolves in the Late Pleistocene and it has been partially replaced by Haplogroup 1 during the Holocene [21]. Patchy distribution of this haplogroup (present in Italy and Eastern Europe, absent from the Iberian Peninsula and the Caucasus) may result from the stochasticity of the lineage replacement process, and bottlenecks that were documented for some European populations [13], [15], [52]. It is likely that the analysis of entire mitochondrial genomes instead of the control region only may reveal additional phylogeographic structure (e.g. see [22] and Figure 2 in [71]). However, this is unlikely to change the conclusion about the distribution of the two haplogroups in Europe, although further subdivisions within each haplogroup may be revealed.

Caucasian wolves share common haplotypes with wolves from both Eastern Europe and the Middle East, consistent with their location between these two geographic regions. Few shared haplotypes between Europe and Asia were identified previously (3 of 47, 6%; [21]). This could have resulted from the large spatial gap between the sampled areas in Europe and Asia, and the data from the Caucasian wolves are partially filling this gap. High percentage of shared haplotypes between the Caucasus and the neighbouring regions suggests that wolf populations from these areas are (or used to be) connected by a considerable level of gene flow.

The microsatellite data also suggest ongoing or recent gene flow between the Caucasian and Eastern European wolf populations. Considerable level of admixture between Caucasian and Bulgarian wolves was detected, suggesting that they are connected by gene flow through intermediary populations (which is possible because of relatively continuous wolf range in the areas between the Caucasus and the Balkans).

We found no evidence for genetic distinctiveness of the Caucasian wolves that would justify their classification as a distinct subspecies *C. l. cubanensis*, which was proposed based on morphological distinctiveness. However, we cannot exclude that environmental differences between predominantly mountainous habitats of the Caucasus and lowland habitats of the neighbouring regions (e.g. European Russia) are associated with some level of genetic discontinuity between wolf populations. Such discontinuities between regions differing in types of habitat and potential prey were reported for the grey wolves elsewhere (e.g. [26], [72]–[74]).

Our results did not provide an unambiguous answer on the question whether the Caucasus played a role of a glacial refugium for the grey wolf. We found no evidence for the demographic expansion in the Caucasian population based on mtDNA mismatch distribution and Tajima’s D and Fu’s Fs tests. However, the mismatch distribution is consistent with the spatial expansion model, which may indicate that the Caucasus might have served as a source population for the neighbouring areas in the past. Comprehensive sampling of these neighbouring areas is needed to better understand the evolutionary history of wolves in this region.

### Population Structure and Inbreeding Levels in the Caucasian Wolves

Despite considerable geographic distance and habitat differences between Georgia and Nagorno-Karabakh, we did not find evidence for their differentiation at nuclear microsatellite loci, and differentiation at mtDNA was only detected when applying a spatially explicit model. The difference between the patterns at nuclear versus mitochondrial markers may be due to higher effective population sizes for microsatellite loci as compared with mtDNA, or due to potentially longer dispersal distances by males. However, there is no unequivocal evidence for male-biased dispersal and gene flow in wolves. Dense sampling is needed to identify weak population differentiation at microsatellite loci (e.g. [26], [74]), and our sample size might have been too small for this purpose. On the other hand, strong population differentiation is clearly detectable even for small sample sizes. For example, in the present study, Spanish wolves were clearly distinguished from both Bulgarian and Caucasian wolves, although only seven individuals from Spain were included in the analysis. Therefore, our study shows that there is no strong population differentiation within the south Caucasus, but mtDNA data suggest that there may be cryptic, fine scale differentiation.

Genetic variability at microsatellite loci in the Caucasian wolves was comparable to the Bulgarian wolves, and higher than in Italian and Spanish wolves (Table 3). Inbreeding coefficient F_IS_ in the Caucasus was lower compared with Bulgaria, where high inbreeding levels were attributed to intense hunting that destabilises social structure of wolf packs [27]. In Georgia, there are legal restrictions on wolf hunting, and in the entire southern Caucasus, wolves are being hunted mostly in areas well-populated by humans, in particular as a response to the incidents of predation on livestock. Hunting is infrequent higher in the mountains, and therefore its overall effect may be smaller. This may explain lower F_IS_ in the Caucasus as compared with Bulgaria. On the other hand, effective population sizes are similarly small in these two areas. In Bulgaria, small effective size could be explained by both inbreeding and documented population bottleneck that took place in the 1970s. We found no clear evidence for recent bottleneck in the Caucasus based on the Bottleneck test. However, the genetic test for the Bulgarian population, where substantial reduction of the population size in 1970s is well documented, gave similarly inconclusive results.

### Long-term Demographic Patterns Inferred from Genome-wide SNP Data

Estimated heterozygosity levels in the Caucasian wolves (H_O_ = 0.217, H_E_ = 0.239) were similar to those in the Balkans (H_O_ = 0.217, H_E_ = 0.223 [15]), and similar to or lower than those in other Eastern European populations (H_O_ = 0.214–0.235, H_E_ = 0.219–0.263 [15]; H_O_ = 0.242–0.292, H_E_ = 0.250–0.292 [55]). However, the Caucasian wolves had higher heterozygosity as compared with isolated populations from Italy (H_O_ = 0.165, H_E_ = 0.174 [55]) and Spain (H_O_ = 0.173, H_E_ = 0.169 [15]) that experienced long-term bottlenecks (i.e., low population numbers over long periods [15]). Relatively low heterozygosity level as compared with Eastern European wolves may result from the fact that it was assessed for only four individuals sampled from one region of the Caucasus. However, even such small sample proves that the heterozygosity in Caucasian wolves is higher in comparison with Italian and Spanish wolves, which implies that their variability has not been substantially reduced by long-term demographic declines and isolation, as it occurred in the Apennine and Iberian Peninsulas [13], [15], [52].

Consistently, LD level (quantified as the distance for which *r* ^2^ decays below 0.5) was moderate (7.5 Kb) and within the range observed for the local populations from Eastern Europe (2.5–10 Kb), in contrast to the high LD observed in the Iberian Peninsula (275 Kb) and Italy (>1,000 Kb) [15] (Figure 4B). However, predominance of long ROH segments (>1 M) over shorter ROH segments suggests that there is some level of inbreeding in the contemporary population, while in the past inbreeding occurred less frequently (see [57]). Similar patterns were observed in Eastern European wolf populations, and it differentiated them from the Italian and Iberian populations that have maintained low population sizes for many generations [15] (Figure 4A).

The demographic reconstruction based on LD patterns showed a progressive decline of the Caucasian population from about 60,000 years ago until present (Figure 4C). Similar reconstruction showed declines in other wolf populations from Eastern Europe, Italy and Spain [15]. Long-term population declines (from about 20,000 years ago until present) were also inferred for wolf populations in Europe, Middle East and East Asia based on whole-genome sequence data [75]. This result shows that the long-term demographic trend in the Caucasus was consistent with the trends in the neighbouring European and Middle-Eastern populations. The most recent estimate of *N_E_* = 144 (95% CI 140–148) at about 150 years ago is within the range of the confidence intervals for the microsatellite-based *N_E_* estimates for the contemporary population (68–159), consistent with the lack of a recent bottleneck.

## Conclusions

We found that grey wolves in the Caucasus have high genetic diversity at all types of markers analysed as compared with wolf populations from Southern Europe. All Southern European populations considered went through a genetic bottleneck of different severity and duration, but we found no evidence for such event in the Caucasian population, which may explain its higher diversity. On the other hand, Caucasian wolves had relatively low nucleotide diversity at mtDNA sequences, which may be explained by the presence of only one of the two main mtDNA haplogroups occurring in Eurasian wolves.

Caucasian wolves share mtDNA haplotypes with both Eastern European and Middle Eastern wolves, suggesting past or ongoing gene flow. Microsatellite data also suggested some level of connectivity between the Caucasus and the Balkans through intermediary populations. Our results do not support the classification of Caucasian wolves as a distinct subspecies *C. l. cubanensis*, which was proposed based on morphological distinctiveness. However, it should be stressed that weak fine-scale genetic differentiation may remain undetected for small sample sizes, and we were unable to compare Caucasian wolves with their nearest neighbouring populations.

Similar as other grey wolf populations from Europe and the Middle East, Caucasian wolves show evidence for admixture with domestic dogs. However, the level of admixture is moderate and – at least on the short term – it does not seem to affect the genetic or ecological integrity of the wolf population, i.e. the genetic and ecological distinction between the two species is unambiguous. Although the Caucasian wolves displayed high genetic variability and relatively low levels of inbreeding, they were affected by other conservation problems that occur in many other wolf populations, such as low effective population sizes and the occurrence of hybrids. Therefore, this population requires further genetic monitoring, as well as ecological studies that would allow us to better understand the role of the grey wolf in the ecosystems of the Caucasus and their vulnerability to environmental changes.

## Supporting Information

File S1 **Supporting text:** Description of the study area; Null allele detection; A comment on the relationship between a population bottleneck and inbreeding; Differentiating between mtDNA haplotypes of grey wolves and domestic dogs (a comment to Table 2); **Figure S1**, Admixture levels in individuals identified as grey wolves based on morphology, inferred based on STRUCTURE analysis; **Figure S2**, Graphical representation of the assignment of individual wolves to the Caucasian, Bulgarian and Spanish populations; **Figure S3,** Observed mismatch distribution for mtDNA control region sequences of grey wolves from the Caucasus, in comparison with the expected distribution for demographic expansion model and spatial expansion model.; **Figure S4,** Population structure in the southern Caucasus inferred from mtDNA haplotype distribution using the spatially explicit model implemented in GENELAND; **Figure S5,** Linkage disequilibrium and demographic patterns in the Caucasian wolves inferred from genome-wide SNP data, with the confidence intervals assessed based on bootstrap analysis.
(PDF)
