# Supplementary material for: Genetic Variability of the Grey Wolf Canis lupus in the Caucasus in Comparison with Europe and the Middle East: Distinct or Intermediary Population?
Source: PLoS One. 2014 Apr 8;9(4):e93828. doi: 10.1371/journal.pone.0093828 (PMC3979716; doi:10.1371/journal.pone.0093828)

SUPPLEMENTARY INFORMATION

Genetic variability of the grey wolf *Canis lupus* in the Caucasus in comparison with Europe and the Middle East: Distinct or intermediary population?

Description of the study area

Georgia is located in the west of the South Caucasus region on the southern slopes of Great Caucasus Mountain Range (Figure 1B), and covers an area of 69,500 square km. Two thirds of the country is mountainous - the average height is 1,200 m above sea level (a.s.l.), and the largest height is 5,184 m a.s.l. Mountains dominate the northern, central and southern parts of the country: the Great Caucasus in the north, the Likhi range in the central part and the lesser Caucasus in the south. To the west, the Colchis lowland plains extend to the Black Sea, and the Iberia Plains in the east open to the Caspian basin. The climate of Georgia is similarly diverse: West Georgia is characterized by a relatively humid subtropical climate, while East Georgia has a drier, moderately humid climate. The climatic differences of east and west Georgia account for a major contrast in ecosystem diversity, and vertical zonation between the two areas. The main biomes are: forest, subalpine zone, alpine zone, subnival zone, nival zone and semi-deserts, and in East Georgia steppe and arid light woodlands. Average human density in Georgia is 65.4/km^2.

Most wolf samples analysed in this study originated from three regions: Kazbegi, Svaneti and Colchis. Kazbegi region is a part of the Central Great Caucasus mountain massif in Eastern Georgia, with the average elevation of 2,859 m a.s.l. Natural landscapes of this region vary from deep canyons with fast mountain streams and subalpine vegetation to high peaks covered with glaciers. Svaneti region is a part of the Central Great Caucasus in north-western Georgia, with the average elevation of about 1,800 m a.s.l. The landscape is dominated by mountains separated by deep gorges. The zone below 1,800 m a.s.l. is covered by mixed and coniferous forests. The zone between 1,800 and 3,000 m a.s.l. consists of alpine meadows and grasslands, and high peaks are covered with glaciers. Colchis (Kolkheti) lowland is situated east of the Black Sea Coast. The elevation ranges between 0 and 22 m a.s.l. The dominating natural landscapes of Colchis are temperate forests and wetlands.

Nagorno-Karabakh is located at the southeastern range of the Lesser Caucasus Mountains (Figure 1). Most of the region is mountainous, and human density is low - 29/km^2. Most wolf samples were collected from three regions: Martakert, Kashatakh

and Hadrut. Martakert region has an average elevation of 1,200–1,450 m a.s.l. and its natural vegetation varies from deciduous, coniferous and mixed forest to subalpine and alpine vegetation. This region is characterised with abundance of ungulates and carnivorous mammals, including wolves, jackals, bears, and lynx. Kashatakh region includes mountainous areas covered with forest, high mountain peaks of 3,000–3,500 m a.s.l., covered by snow, and semidesert and desert zones with average heights 550–660 m a.s.l. Hadrut region includes both mountainous and lowland areas, with average height in different parts varying from 620 to 1,050 m a.s.l. The north-east part of the region is mountainous, with vegetation zones from the forest to alpine vegetation, and highest peaks reaching 2,300–2,500 m a.s.l. In the southern part there are lowlands of the river Araks, covered by meadows and pastures, which attract wild ungulates and carnivores. It is the area with highest concentration of livestock farms in Nagorno-Karabakh. However, even in the lowland areas human density is relatively low, and there are large areas uninhabited by humans.

Null allele detection

To test for the presence of null alleles at the microsatelite loci analysed in this study, we used four programs: CERVUS 3.0 (Summers & Amos 1997), MICRO-CHECKER 2.2.1 (van Oosterhout et al. 2004), ML-NullFreq (Kalinowski & Taper 2006) and GENEPOP (Rousset 2008). For ML-NullFreq we performed 100,000 randomizations and we used both null alleles frequency and *P*-value as a measure of null alleles in a locus. The presence of null alleles was assessed in each of the three populations (Caucasus, Bulgaria and Spain) separately.

The number of loci with detected null alleles varied among the populations and the detection methods. GENEPOP indicated the highest frequency of occurrence of the null alleles, and MICRO-CHECKER the lowest. For true null alleles, consistency among methods and populations should be expected, and therefore this result indicated that these putative null alleles were false positives resulting from deviations from the Hardy-Weinberg equilibrium in the populations assessed (see Dąbrowski et al. 2014). Null alleles were detected most frequently in the Bulgarian population, where a significant population structure and high inbreeding coefficient F_{IS} were earlier detected (Moura et al. 2013). Moreover, the number of loci with putative null alleles detected within the two Bulgarian subpopulations was much smaller as compared to the entire population, and the loci with putative null alleles were

inconsistent between the subpopulations (Moura et al. 2013). In this study, we also found population structure and high F_{IS} in the Caucasian population. The putative null alleles detected are likely to be due to the heterozygote deficit resulting from population structure and inbreeding, as each of the detection methods assumes Hardy-Weinberg equilibrium in the populations assessed (see Dąbrowski et al. 2014). Therefore, we concluded that the observed pattern does not justify the exclusion of any locus from the data analysis due to the presence of putative null alleles.

A comment on the relationship between a population bottleneck and inbreeding

Populations that went through a bottleneck and then increased in size do not have to show signs of a recent inbreeding, i.e. there may be no cases of breeding between close kin, even though average relatedness in a population is high. This is the case of Italian wolves: the population went through a strong, long term bottleneck, but the genetic patterns suggest that now breeding between kin is rare there: the Italian population has a very extensive linkage disequilibrium, but low proportion of long ROHs compared to short ROHs (Pilot et al. in press) (see Figure 4 in the main manuscript).

On the other hand, inbreeding may occur without an extensive bottleneck, e.g. if a population is extensively hunted – examples of such cases were described e.g. in Jędrzejewski et al. (2005) and Moura et al. (2013). Such populations do not have extensive linkage disequilibrium, but have high proportion of long ROHs. The example is a local population from North Belarus (Pilot et al. in press), and the Caucasian population also follows this pattern (Figure 4). Finally, populations that went through recent reductions in population size (e.g. Mexican wolves) or populations established by few founders (e.g. Isle Royale wolves) have extensive linkage disequilibrium as well as high proportion of long ROHs (vonHoldt et al. 2011).

Differentiating between mtDNA haplotypes of grey wolves and domestic dogs (a comment to Table 2)

The issue of differentiating between wolf and dog haplotypes is problematic for the following reasons:

- The split between the two species (or subspecies according to the present taxonomic classification) was very recent in the evolutionary timescale, and therefore they may still share a number of common ancestral haplotypes.
- It is likely that after the initial domestication event some level of gene flow was maintained between the two (sub)species, so mtDNA haplotypes could have been exchanged.
- Consistent with the two earlier points, phylogenetic studies on wolf and dog mtDNA haplotypes (e.g. Vilá et al. 1997, Savolainen et al. 2002, Verginelli et al. 2009) show that these two (sub)species are not reciprocally monophyletic in mtDNA, the dog clades include wolf haplotypes, and some haplotypes are shared between dogs and wolves.
- There are accounts from various parts of the word (including the Caucasus) of deliberate crossing of dogs with wolves by humans to "improve the breed" (Kopaliani et al. in press).
- Contemporary hybridisation has been documented in different regions of Eurasia (including the Caucasus), with backcrossing into both wolf and dog populations (see references in the main text). Therefore, mtDNA haplotypes could have been recently exchanged.

For these reasons, distinguishing between wolf and dog haplotypes is problematic. However, in the case of the haplotypes found in the Caucasus, we identified only one GenBank match with the domestic dog haplotypes, so the remaining haplotypes may be assumed to derive from wolves.

References

Dąbrowski MJ, Pilot M, Kruczyk M, Żmihorski M, Umer HM, Gliwicz J (2014) Reliability assessment of null allele detection: inconsistencies between and within different methods. Molecular Ecology Resources 14: 361–373.

Jędrzejewski W, Branicki W, Veit C, Medugorac I, Pilot M, et al. (2005) Genetic diversity and relatedness within packs in an intensely hunted population of wolves *Canis lupus*. Acta Theriologica 50: 3–22.

Kalinowski ST, Taper ML (2006) Maximum likelihood estimation of the frequency of null alleles at microsatellite loci. Conservation Genetics 7: 991–995.

Kopaliani N, Shakarashvili M, Gurielidze Z, Qurkhuli T, Tarkhnishvili D (in press) Gene flow between wolf and shepherd dog populations in Georgia (Caucasus). Journal of Heredity.

Moura AE, Tsingarska E, Dąbrowski M, Czarnomska SD, Jędrzejewska B, Pilot M (2013) Unregulated hunting and genetic recovery from a severe population decline: the cautionary case of Bulgarian wolves. Conservation Genetics, DOI: 10.1007/s10592-013-0547-y.

Pilot M, Greco C, vonHoldt BM, Jędrzejewska B, Randi E, et al. (in press) Genome-wide signatures of population bottlenecks and diversifying selection in European wolves. Heredity.

Rousset F (2008) Genepop’007: a complete re-implementation of the Genepop software for Windows and Linux. Molecular Ecology Resourses 8: 103–106.

Savolainen P, Zhang YP, Luo J, Lundeberg J, Leitner T (2002) Genetic evidence for an East Asian origin of domestic dogs. Science 298: 1610–1613.

Summers K, Amos W (1997) Behavioral, ecological, and molecular genetic analyses of reproductive strategies in the Amazonian dart-poison frog, *Dendrobates ventrimaculatus*. Behavioral Ecology 8: 260–267.

van Oosterhout C, Hutchinson WF, Wills DMP, Shipley P (2004) MICRO-CHECKER: software for identifying and correcting genotyping errors in microsatellite data. Molecular Ecology Notes 4: 535–538.

Verginelli F, Capelli C, Coia V, Musiani M, Falchetti M, et al. (2005) Mitochondrial DNA from prehistoric canids highlights relationships between dogs and south-east European wolves. Molecular Biology & Evolution 22: 2541–2551.

Vilà C, Savolainen P, Maldonaldo JE, Amorim IR, Rice JE, et al. (1997) Multiple and ancient origins of the domestic dog**.** *Science* 1997, 276:1687-1689.

vonHoldt BM, Pollinger JP, Earl DA, Knowles JC, Boyko AR, et al. (2011) A genome-wide perspective on the evolutionary history of enigmatic wolf-like canids. Genome Research 21: 1213–1222.

Figure S1. Admixture levels in individuals identified as grey wolves based on morphology, inferred based on STRUCTURE analysis. Colours in the pie charts represent assignments (q-values) to genetic clusters corresponding to three canid species. Assignment values ≤0.01 are not presented on the pie charts.

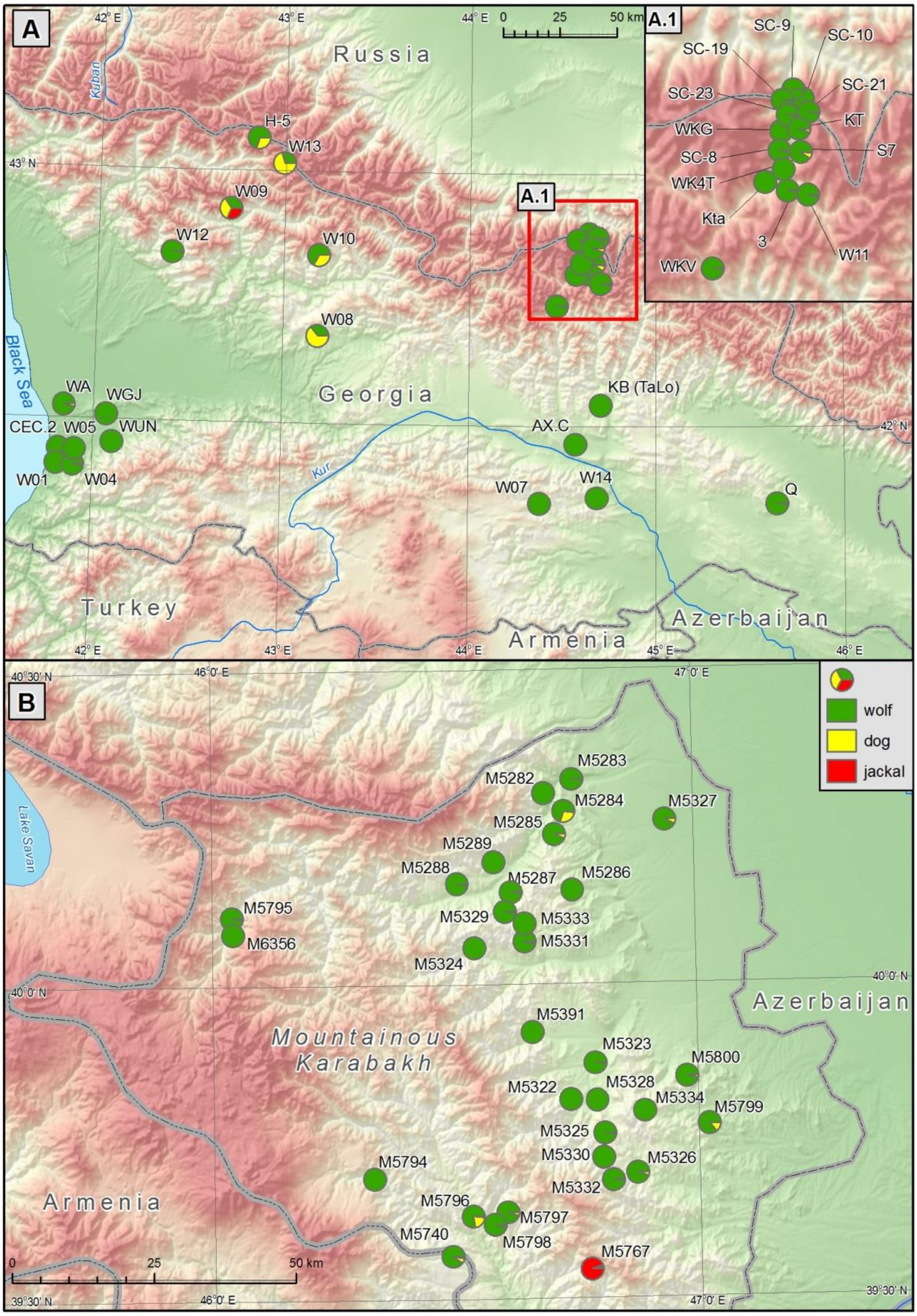

Figure S2. Graphical representation of the assignment of individual wolves to the Caucasian, Bulgarian and Spanish populations. A) GENALEX assignment test; B) STRUCTURE analysis without prior population information (K=3); C) STRUCTURE analysis with prior population information (K=3).

A

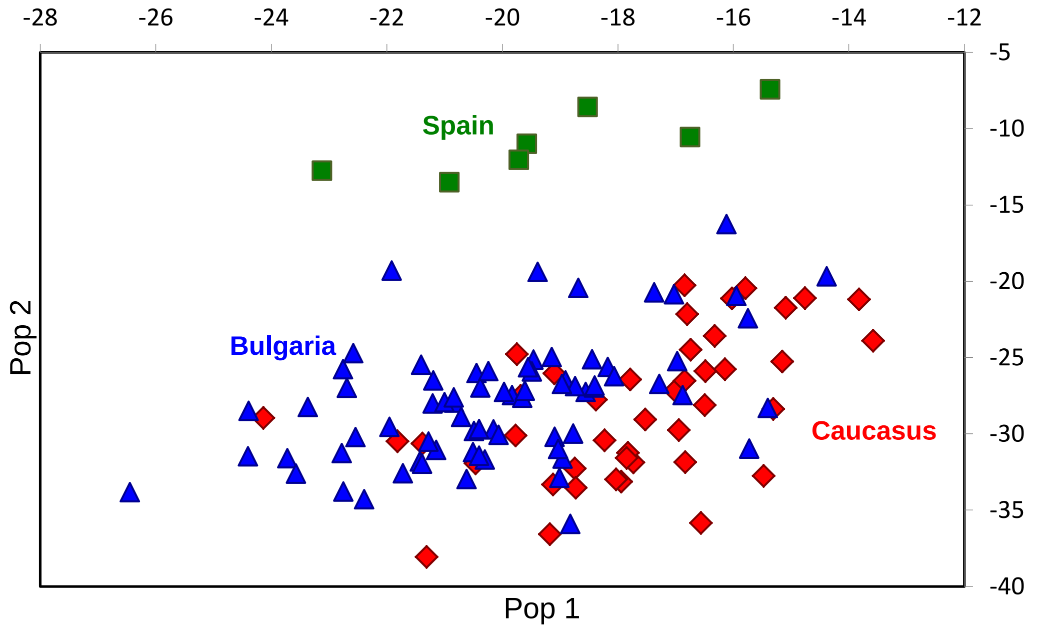

B

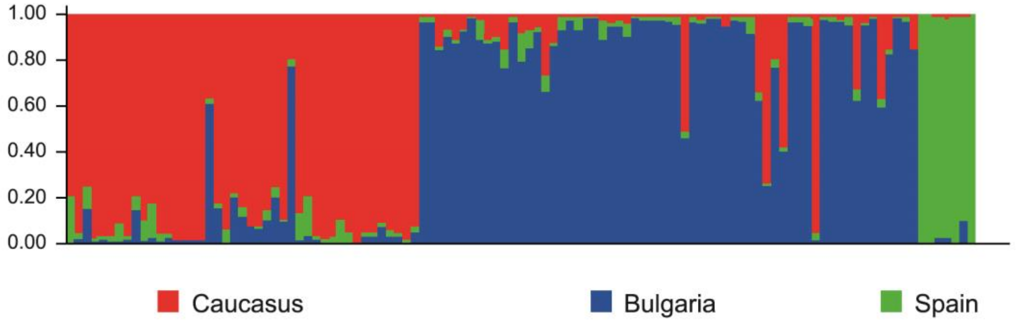

C

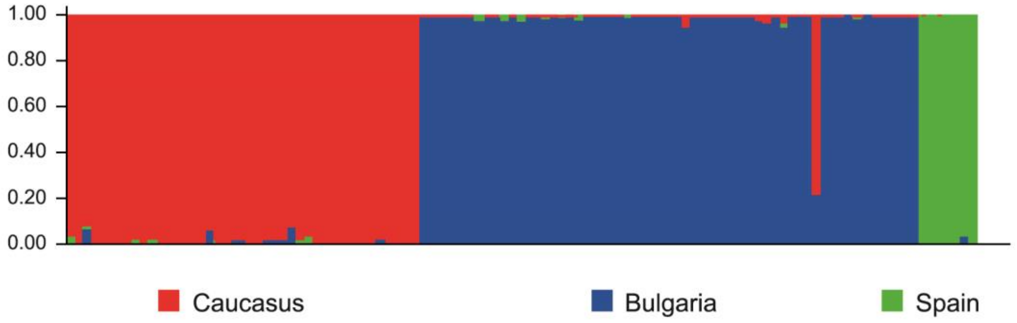

Figure S3. Observed mismatch distribution for mtDNA control region sequences of grey wolves from the Caucasus, in comparison with the expected distribution for (A) demographic expansion model and (B) spatial expansion model.

A

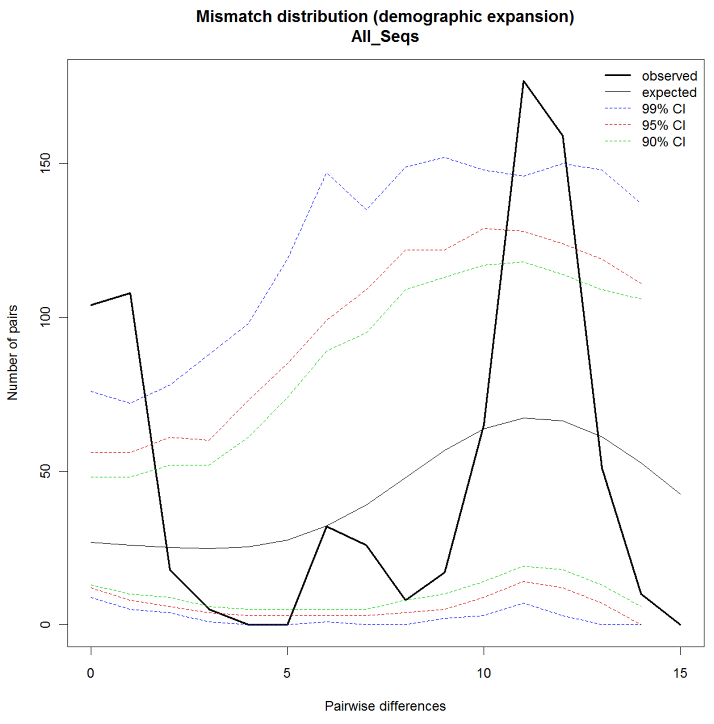

B

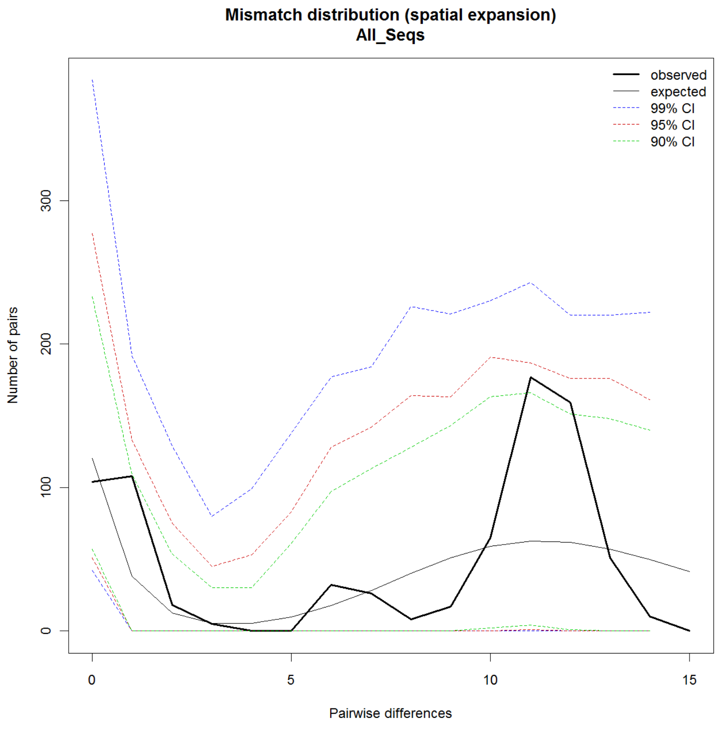

Figure S4. Population structure in the southern Caucasus inferred from mtDNA haplotype distribution using the spatially explicit model implemented in GENELAND. Two genetic clusters were detected, and the figure represents spatial distribution of posterior probability for individuals to belong to Cluster 1.

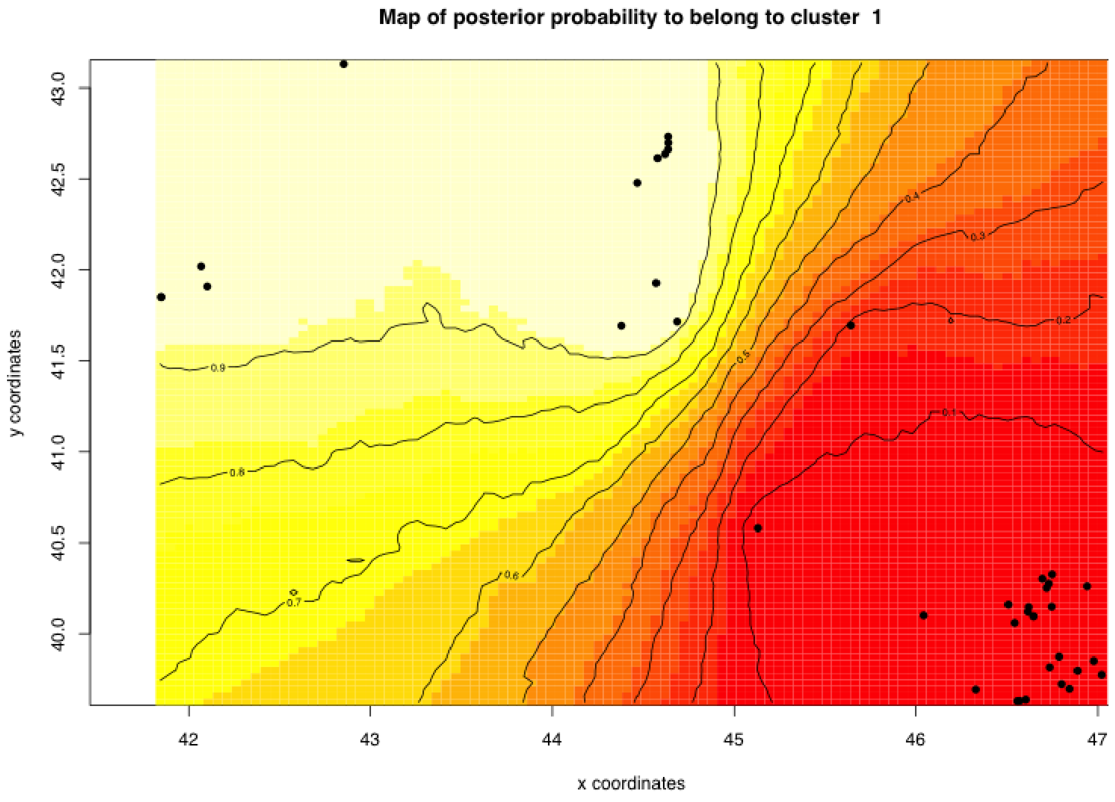

Figure S5. Linkage disequilibrium and demographic patterns in the Caucasian wolves inferred from genome-wide SNP data, with the confidence intervals assessed based on bootstrap analysis. (A) Extent of linkage disequilibrium, represented as changes in an average genotypic association coefficient r^2 with an increasing inter-SNP distance. (B) Temporal changes of effective population size (N_E).

A

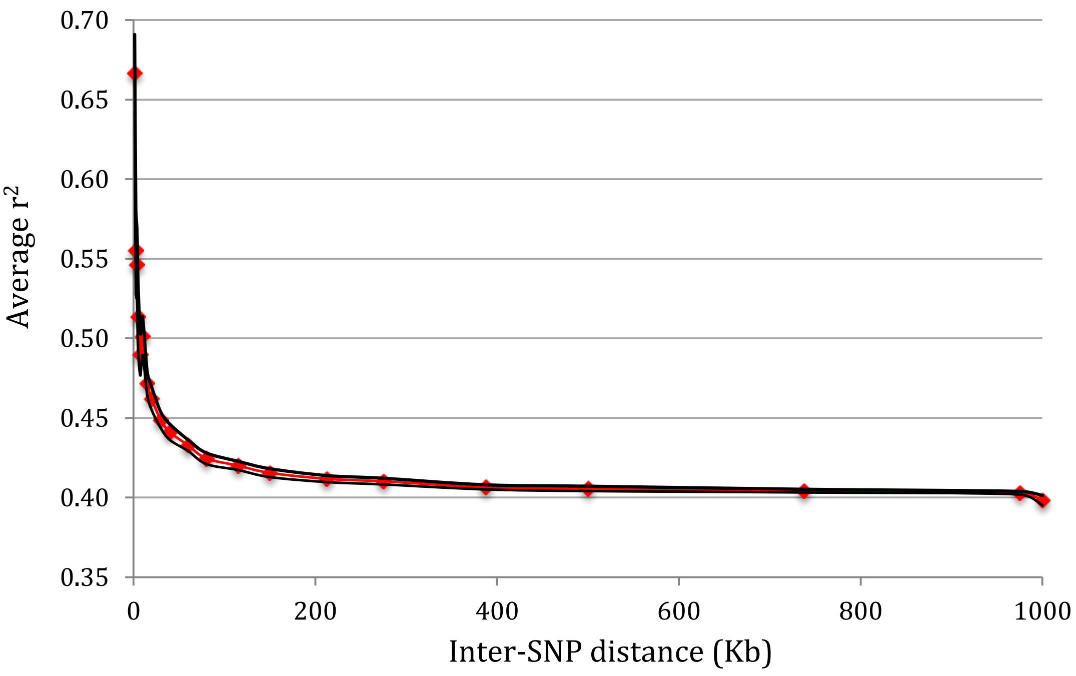

B

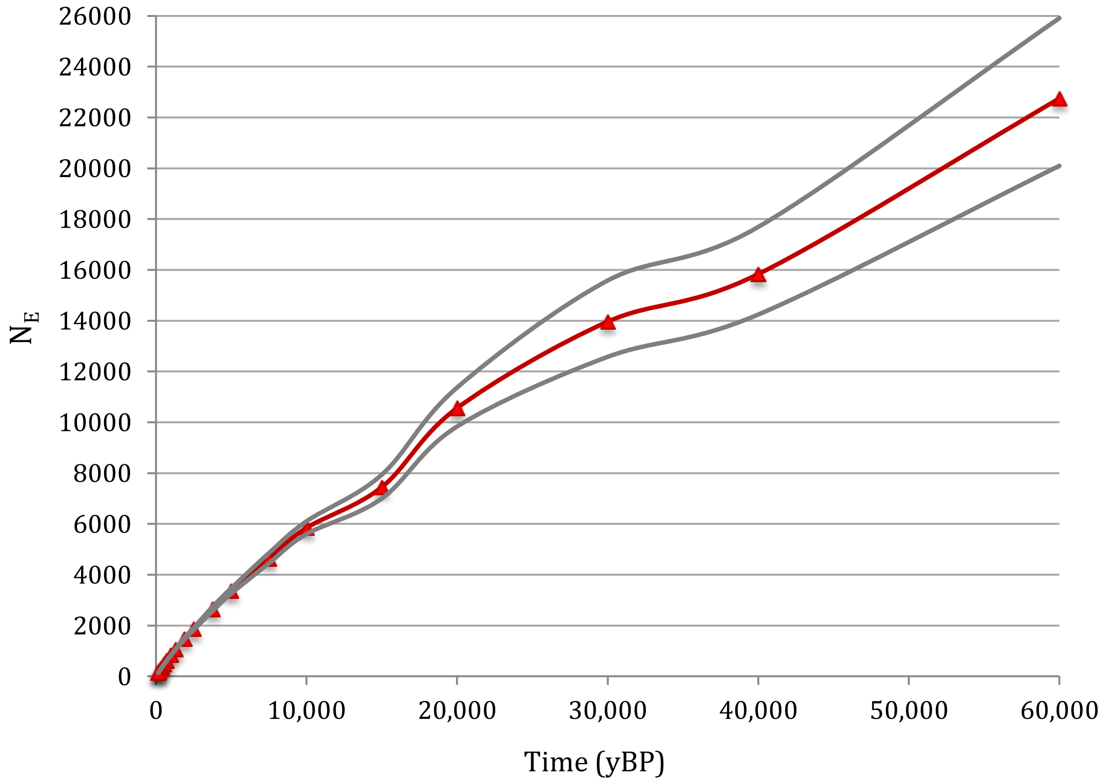

Supplement: File S1 — Supporting text: Description of the study area; Null allele detection; A comment on the relationship between a population bottleneck and inbreeding; Differentiating between mtDNA haplotypes of grey wolves and domestic dogs (a comment to Table 2); Figure S1, Admixture levels in individuals identified as grey wolves based on morphology, inferred based on STRUCTURE analysis; Figure S2, Graphical representation of the assignment of individual wolves to the Caucasian, Bulgarian and Spanish populations; Figure S3, Observed mismatch distribution for mtDNA control region sequences of grey wolves from the Caucasus, in comparison with the expected distribution for demographic expansion model and spatial expansion model.; Figure S4, Population structure in the southern Caucasus inferred from mtDNA haplotype distribution using the spatially explicit model implemented in GENELAND; Figure S5, Linkage disequilibrium and demographic patterns in the Caucasian wolves inferred from genome-wide SNP data, with the confidence intervals assessed based on bootstrap analysis. (PDF) [file pone.0093828.s001.pdf]
